# A young man with exertional chest discomfort

**DOI:** 10.1007/s12471-019-1276-8

**Published:** 2019-04-11

**Authors:** L. E. Lezcano Gort, B. Roque Rodríguez, M. R. Porro Fernández

**Affiliations:** Department of Cardiology, San Pedro de Alcantara Universitary Hospital, Cáceres, Spain

## Answer

**Fig. 1:**
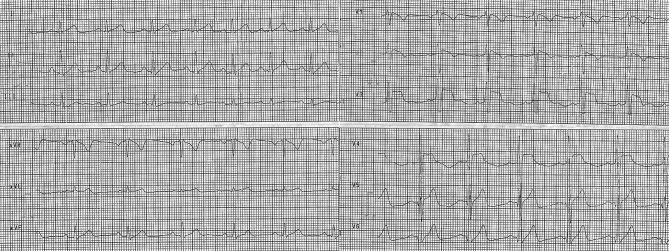
Out-of-hospital ECG at first medical contact

Out-of-hospital electrocardiogram (ECG) shows a sinus rhythm of 85 beats per minute (bpm), narrow QRS complex, and ST-segment elevations in leads V1–V4 (coved morphology in V1–V2), with a terminal negative T wave in V1–V2 (Fig. 1). ECG findings could be suspicious for acute anteroseptal myocardial infarction, but reciprocal ST-segment changes are lacking. The ECG is also suggestive of a coved-type Brugada-like pattern, and the patient had no history of syncope or any other cardiac symptom, nor a family history of unexplained sudden cardiac death.

**Fig. 2:**
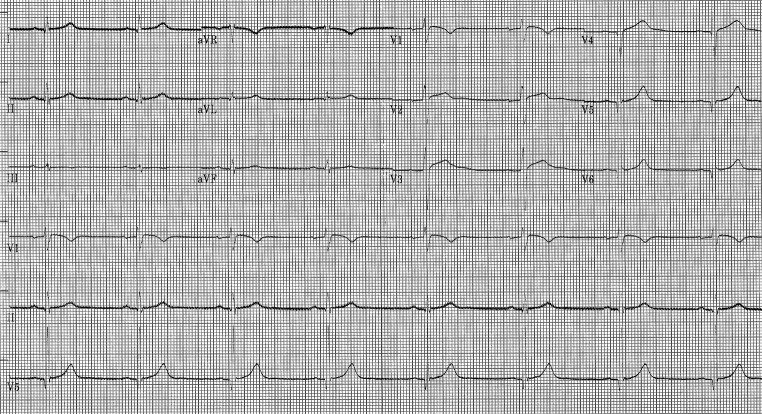
ECG obtained in the Intensive Care Unit

The patient met criteria for exertional heat stroke [1], and was admitted to the intensive care unit for rhythm monitoring. Fluid therapy was started, and within the first 2 hours the ECG showed sinus bradycardia of 48 bpm, normalised ST segment, QTc of 430 ms, and prominent U waves in V2–V3 (Fig. 2), remaining with similar features during the hospital stay. Echocardiogram and computed tomography coronary angiography performed before discharge were normal.

Diffuse ST-T deviations have been described in patients with heat stroke [2], but right precordial leads ST elevation suggestive of Brugada pattern in this context is exceptional [3]. Ion channels sensitive to temperature may explain this electrocardiographic finding in susceptible individuals. In the present case, the rapid resolution of ECG abnormalities favours the diagnosis of Brugada-like ECG pattern induced by exertional heat stroke, and a negative ajmaline provocation test reinforces the diagnosis.

## Conclusion

Type 1 Brugada-like ECG pattern induced by exertional heat stroke.

## References

[1] Hifumi T, Kondo Y, Shimizu K, Miyake Y (2018). Heat stroke. J Intensive Care.

[2] Mimish L (2012). Electrocardiographic findings in heat stroke and exhaustion: A study on Makkah pilgrims. J Saudi Heart Assoc.

[3] Lacunza J, San Román I, Moreno S (2009). Heat stroke, an unusual trigger of Brugada electrocardiogram. Am J Emerg Med.

